# Occult Central Lymph Node Metastasis in cN0 Papillary Thyroid Carcinoma Patients Undergoing TOETVA Procedure

**DOI:** 10.1155/2023/4779409

**Published:** 2023-11-30

**Authors:** Hau Nguyen Xuan, Tu Do Anh, Hien Nguyen Xuan, Duong Pham Thai, Quang Le Van

**Affiliations:** ^1^Department of Oncology, Hanoi Medical University, No. 1 Ton That Tung Street, Dong Da, Hanoi, Vietnam; ^2^Department of Oncology and Palliative Care, Hanoi Medical University Hospital, No. 1 Ton That Tung Street, Dong Da, Hanoi, Vietnam; ^3^Vietnam National Cancer Hospital, Tuu Liet, Tam Hiep, Thanh Tri, Hanoi, Vietnam

## Abstract

**Background:**

In this study, we evaluate the rate of CLNM and related factors in patients with cN0 PTC undergoing transoral endoscopic thyroidectomy vestibular approach (TOETVA), a feasible and safe procedure that is widely approved for early stage PTC patients.

**Method:**

A cross-sectional study was performed on 346 patients who underwent TOETVA due to thyroid cancer in the Department of Oncology and Palliative Care, Hanoi Medical University Hospital, from January 2020 to December 2021. The clinical, surgical, and pathological characteristics were recorded.

**Results:**

The mean age was 36.1 ± 9.1 (13–67) years. Females accounted for 96%. Total thyroidectomy was applied in 55 cases (15.9%), and conservative thyroidectomy accounted for 291 (84.4%). The median number of harvested lymph nodes in ipsilateral and bilateral CND groups is 5 (IQR: 3–7) and 7 (IQR: 3–10). The median number of metastasized lymph nodes in these two groups is 2 (IQR: 1–3) and 3 (IQR: 2–6), respectively. The rate of CLNM was 39.9%. Thyroiditis increased the number of harvested lymph nodes: 8.3 ± 0.7 (1–24) nodes, *p* = 0.002. Tumor size on ultrasound, young age (<29 years old), and stage of tumor increased the possibility of CLNM, *p* < 0.05. Univariate and multivariate logistic regression revealed that young age (<29 years old) and gross tumor invasion were independent risk factors of high number of CLNM with *p* < 0.05.

**Conclusion:**

In summary, CLNM rate in patients with cN0 PTC accounted for 39.9%. With the facilities of pCND by TOETVA, a procedure that is widely approved for early PTC and has excellent cosmetics and oncological results, pCND should be considered in patients with risk factors like young age or large tumor. High volume of CLNM is associated with young age and gross tumor extension, and total thyroidectomy should be indicated in these patient groups.

## 1. Introduction

With the development of imaging tests such as ultrasound or CT scans and the ability to perform guided biopsies in the office setting, most PTC patients are now diagnosed with early stage, and many cases even have clinically lymph node negative. The prognosis of this patient group is excellent. Microscopic metastatic lymph node is defined by American Thyroid Association (ATA) in 2015 as a positive node with a diameter less than 2 mm, and this phenomenon was observed in postoperative pathological results very frequently, to be between 20.7% and 62% [1, 2]. This condition is an important prognostic factor that increases local recurrent rate and may alter post-surgery treatment strategy, and sometimes even a re-operative surgery is needed [2–5]. This has been discussed in many previous studies, and now we have more experience dealing with this problem and prophylactic central neck dissection (pCND) might be the key to solve. pCND has the potential to lower recurrence rates, improve the accuracy of staging, predict risk for recurrent, reduce serum thyroglobulin levels postoperatively leading to more effective detection of persistent or recurrent disease, and provide a survival benefit in some patients and can guide the use of RAI therapy [6–9]. As a result, the need for re-operative surgery in the central neck is reduced, as well as the risks of injury to the recurrent laryngeal nerves (RLNs) and parathyroid glands during the second surgery. Transoral endoscopic thyroidectomy vestibular approach (TOETVA) has been used in our department at Hanoi Medical University Hospital since 2018 and has many advantages, such as good cosmetic results and effective central node dissection, including giving access to both thyroid lobes via a single subcutaneous dissection route, providing surgeons with excellent exposition and a straight path to the central part of the neck, and facilitating us to perform CND, especially prophylactic CND [10–12]. Early results of this procedure in the thyroid cancer patients group show that the local recurrence rate is low [13]. Therefore, we conducted this study to evaluate CLNM status and related factors in patients with cN0 PTC undergoing TOETVA, which may help surgeons to optimize their choice during operation.

## 2. Method

### 2.1. Patients

This cross-sectional study included a total of 346 cN0 papillary thyroid carcinoma patients who underwent TOETVA procedure in Department of Oncology and Palliative Care, Hanoi Medical University Hospital, from January 1, 2020, to December 12, 2021. The surgical procedure of TOETVA was described in our previous study on thyroid microcarcinoma [14, 15]. Selection criteria include all of the following: (1) diagnosed with papillary thyroid cancer, (2) tumor size less than 2 cm, (3) noninvasive tumor confirmed by examination and ultrasound, and (4) no lymph node detected or nodes smaller than 2 mm on ultrasound or CT scan images. We excluded patients with lymph node involvement by preoperative and intraoperative assessment, diagnosed with other types of thyroid cancer and clinical invasive thyroid tumors. Thyroid lobectomy and isthmectomy with prophylactic ipsilateral CND (level VI and VII lymph nodes) were indicated in patients with unilateral cT1 tumors. In the T3b-intraoperative and bilateral or isthmus tumor group, total thyroidectomy and bilateral prophylactic CND were indicated.

We evaluated the clinical parameters age, gender, Graves' disease history, tumor size, the external extension of tumor, the extent of thyroidectomy, coexistent thyroiditis, and number of harvested and metastatic lymph nodes in our study.

### 2.2. Statistical Analysis

To analyze factors that may affect the number of harvested and metastatic status of lymph nodes, 346 patients were subdivided into two groups based on the pathological results of their central lymph nodes. Significant differences in the percentage of variation across different parameters were assessed with the chi-square and Fisher's exact tests. The difference between continuous variables was determined with the independent two-sample *t*-test. Clinicopathological prognostic factors related to the number of harvested or metastatic lymph nodes were identified using univariate and multivariate analyses. Odds ratios (ORs) with a relative confidence interval of 95% (95% CI) were calculated to determine the relevance of all potential predictors of CLNM. Analysis was performed using SPSS software (version 22.0; SPSS, Chicago, IL), and statistical significance was accepted for *p* < 0.05.

## 3. Result

### 3.1. Clinicopathological Parameters

Table 1 illustrates the clinicopathological characteristics of the 346 patients. The mean patient age was 36.1 ± 9.1 years. Three hundred thirty-two female (96%) and fourteen male patients (14%) were included in the study. Four patients (1.15%) have previous history with Graves' disease. The number of patients with cancer in a single lobe is 329 (95.1%), and cancer in the isthmus/both of the thyroid lobes is 17 (4.9%). The majority of these patients received conservative thyroidectomy with 291 patients (84.1%). In patients with ipsilateral CND, median number of harvested lymph node is 5 (interquartile range (IQR): 3–7) and median metastasized lymph node is 2 (IQR: 1–3). In bilateral CND group, the median number of harvested and metastasized nodes is 7 (IQR: 3–10) and 3 (IQR: 2–6), respectively. CLNM was observed in 139 patients (39.9%). The ratio of the number of metastatic lymph nodes to the total number of harvested lymph nodes is 0.5 ± 0.3 (0.05–1). About tumor classification: 298 patients (77.2%) were diagnosed with stage T1a, and 90 patients (26%) had microscopic capsular invasion tumor. Sixty-one patients (17.6%) had coexistent thyroiditis.

### 3.2. Risk Factors for the Number of Harvested Lymph Nodes

Analysis of factors affecting the number of harvested lymph nodes is given in Table 2. Factors such as age, gender, previous history with Graves' disease, tumor size on ultrasound, tumor location, surgical procedure, the extent of CND, T stage, and microscopic capsular invasion status did not affect the number of lymph node harvested in operation with *p* > 0.05. However, the coexisting thyroiditis increases the number of removed lymph nodes with *p*=0.002.

### 3.3. Risk Factors for CLNM

**Figure 1:**
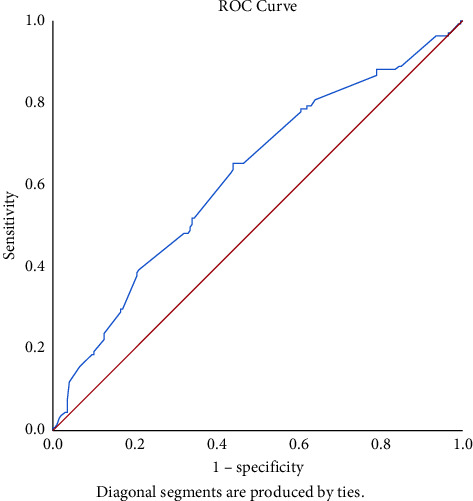
ROC curve analysis of tumor size for predicting CLNM in PTC patients. The cutoff value of the tumor size was 6.5 mm (sensitivity = 0.65, specificity = 0.56, AUC = 0.625, *p*=0.000).

Analysis of factors affecting metastatic status of lymph nodes post-surgery is given in Table 3. Univariate logistic regression demonstrated that factors such as gender, history of Graves' disease, tumor location, thyroiditis status, surgical method, CND on one or both sides, and microscopic capsular invasion status are not associated with the risk of increased lymph node metastasis incident. Larger tumor size, younger age, and higher tumor stage (T1b, T3b) increase the likelihood of lymph node metastasis with *p* < 0.05. To further study the relationship between the tumor size and the incidence of CLNM, we constructed a ROC curve to determine the critical value of this factor for predicting CLNM. As shown in Figure 1, the cutoff value of the tumor size for CLNM was 6.5 mm (sensitivity = 0.65, specificity = 0.56, AUC = 0.625, *p*=0.000).

### 3.4. Risk Factors for High Number of CLNM

**Figure 2:**
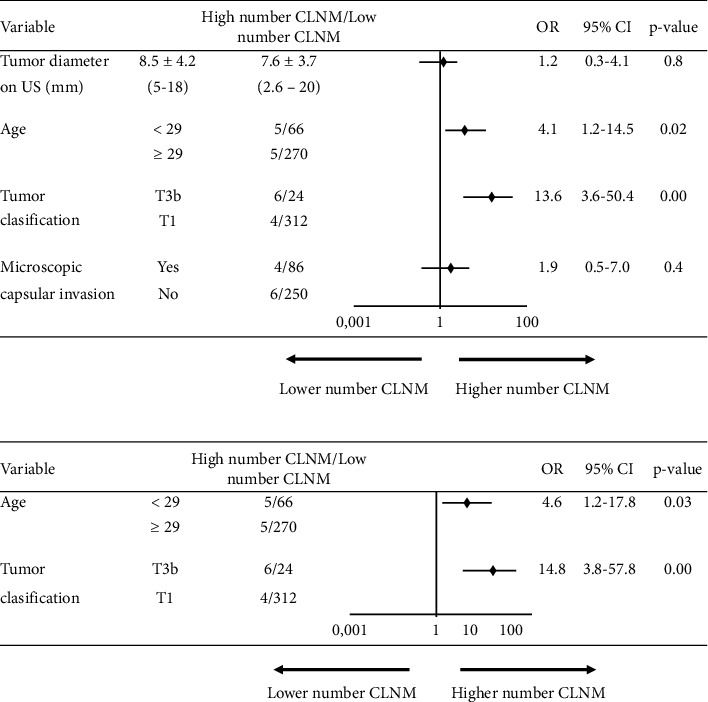
Forest plot of univariate and multivariate logistic regression analyses for risk factors associated with high number of CLNM. (a) Univariate analysis. (b) Multivariate analysis. OR: odds ratio; CI: confidence interval.

Analysis of factors related to the number of microscopic metastasis lymph nodes post-surgery is given in Figure 2. Tumor size on neck ultrasound and thyroiditis status do not increase the number of metastatic nodes. Patients' age at operation younger than 29 years is associated with risk of high number of CLNM with *p*=0.02. Another factor related to the number of metastatic lymph nodes is the tumor stage with *p*=0.00. However, in the subgroup analysis, the number of microscopic metastatic lymph nodes of two groups T1a tumor and T1b tumor was comparable with *p*=0.57, but there is a significant difference between noninvasive tumor and gross invasive tumor groups with *p*=0.002 and OR (95% CI) of 0.1 (0.02–0.3). Multivariate logistic regression revealed that young age (< 29 years) and external extension tumors were independent risk factors for high number (>5) of CLNM.

## 4. Discussion

Nowadays, most PTC patients are diagnosed in early stage, even with clinically lymph nodes negative, and have excellent long-term prognosis [16]. Total thyroidectomy or partial thyroidectomy with or without pCND is the treatment of choice in this patient group, and most decisions about the extent of thyroidectomy and the need of pCND are given in accordance with the surgeon's intraoperative assessment. The indication of pCND in cN0 stage PTC is still controversial. Occult lymph node metastases are considered a very common phenomenon in papillary thyroid carcinoma; this may be found in up to 50% of patients after pCND with no evidence of lymph node metastasis in preoperative assessment [17]. Because of that, adopting pCND in all PTC patients would ideally ensure the eradication of all subclinical metastatic nodes in the central compartment and provide better disease control. Barczyński et al. also demonstrated a significant reduction in both local recurrent rate and disease-specific survival after bilateral pCND in total thyroidectomy patients [9]. In hemithyroidectomy group, a meta-analysis study conducted by Ahn and Kim also showed that pCND significantly reduced the risk of recurrence in the central compartment [18]. Nevertheless, routine central nodal dissection provides more staging information than thyroidectomy alone. Patients with intermediate-risk malignancies may benefit from tailored RAI delivery or more precise dosage estimations based on their nodal status following pCND [19]. Although pCND provides survival benefits and reduces the risk of locoregional recurrence, pCND is also associated with risks of complication, including both temporary and permanent hypoparathyroidism and injury to the recurrent laryngeal nerve. However, this is not so important if the strategy is carried out by high-volume surgeon. For these reasons, we do pCND in almost every early PTC patient by TOETVA with low rate of complications [14]. However, in conservative thyroidectomy procedures with appropriate CND, certain pathologic characteristics of the tumor or lymph nodes revealed after surgery, such as lymph vascular invasion, positive surgical margin, or high number of CLNM, may lead to an indication for completed thyroidectomy. Therefore, the risks of injury to the RLN and parathyroid glands are increased due to the second surgery.

Our study was conducted mainly on women, accounting for 96%. The mean patient age was 36.1 ± 9.1 years. Four patients (1.15%) have previous history with Graves' disease. 95.1% patients have cancer in just one thyroid lobe, while 17 have cancer in the isthmus or both thyroid lobes (4.9%).

The majority of these patients underwent conservative thyroidectomy with 291 patients (84.1%). Postoperative pathological findings show that the new approach to central compartment with TOETVA has good ability in CND: the median number of harvested lymph nodes in ipsilateral and bilateral groups is 5 (IQR: 3–7) and 7 (IQR: 3–10), respectively. Our result is also consistent with the previous study by Ahn and Yi: the number of harvested lymph nodes by TOETVA procedure is 3.19 ± 2.89 and 4.98 ± 3.12 in lobectomy and total thyroidectomy, with no significant difference with open thyroidectomy results [20]. In our investigation of factors influencing the number of lymph nodes harvested, we discovered that certain factors such as age, gender, history of Graves' disease, tumor size on ultrasound, tumor location, the extent of thyroidectomy surgery, central neck dissection (CND) on one or both sides, T staging, and microscopic capsular invasion of the tumor do not have a significant correlation. However, the coexistent thyroiditis condition increases the number of harvested lymph nodes with *p* = 0.002. This result is similar to Song's study in 2017, which shows that the median number of lymph nodes in patients with Hashimoto's thyroiditis group is 11 (8–15) nodes, which is higher than other groups [21].

In postoperative pathological result, we observed that a lot of patients have CLNM: 138 patients out of 346 patients, accounting for 39.9%. Our findings are similar to a study conducted by Eun et al. in 2014, which shows that the frequency of CLNM in patients with cN0 PTC was 36.4% [22]. In a meta-analysis about central lymph node (CLN) metastasis in patients with cN0 PTC conducted by Wei Sun et al., the prevalence of this condition ranged from 11.7% to 63.8% [23]. These results show that CLN metastasis discovered postoperatively in cN0 PTC patients is very common. Because patients diagnosed with PTC have excellent overall survival outcome, especially in early stage with a cause-specific mortality of only 0.3%, our aim of treatment in this patient group is not only about increasing survival rate but also lowering local recurrence rate (LRR) [24]. It is widely known that lymph node metastasis is a proven predictor for LRR in PTC. In patients with recurrence disease, over 80% of these recurrences are found in the central lymph nodes [25–27]. To reduce LRR, pCND is frequently performed by numerous surgeons. In a study conducted by Chen et al. in 2018 about pCND in clinically uninvolved central lymph node metastasis papillary thyroid patients, the results show that patients who underwent pCND had significantly lower LRR but have higher incidence rates of transient RLN injury and transient and permanent hypocalcemia [28]. Thus, it is important to identify the risk factors of CLNM to have an appropriate pCND indication. In our study, we found that gender, Graves' disease history, tumor location, the extent of thyroidectomy surgery, CND on one or both sides, coexistent thyroiditis condition, and microscopic capsular invasion of tumor do not increase the risk of lymph node metastasis. Tumors with microscopic capsular invasion were categorized as T3 in the AJCC TNM 7th classification and total thyroidectomy was advised. However, this group was no longer categorized as a high-risk group in AJCC TNM 8th classification (2017) and only required a partial thyroidectomy. Our study demonstrates that there is no statistically significant difference about CLNM conditions between two groups that have/do not have microscopic capsular invasion in post-op pathology results, with 95% CI: 0.9 (0.6–1.5), *p* = 0.05. This result is consistent with NCCN 2022 guidelines; microscopic capsular invasion is not an indication for total thyroidectomy. Regarding the coexistent thyroiditis condition, our result is similar to that of Song's study in 2017: chronic thyroiditis may increase the number of lymph nodes harvested but does not influence the number of metastatic nodes in PTC. Therefore, even number of lymph nodes harvested during surgery is higher than usual, and a total thyroidectomy procedure may not always be necessary. Surgeons should consider a combination of other factors to determine the appropriate extent of the thyroidectomy for this group of patients [21].

Aside from several factors that do not affect the number of metastatic lymph nodes we mentioned above, univariate logistic regression demonstrated that a larger tumor size on ultrasound increases the risk of metastatic lymph node status with *p* = 0.000. In a study to identify risk factors for neck nodal metastasis in PTC performed on 1066 patients, Zhang et al. reported that a tumor size of greater than 6.5 mm indicated a risk factor for CLNM in papillary thyroid microcarcinoma by using ROC analysis [29]. With the same method, our results showed that the cutoff value of the tumor size for CLNM was 6.5 mm (sensitivity = 0.65, specificity = 0.56, AUC = 0.625, *p* = 0.000). Therefore, we consider that tumor size on US >6.5 mm could be used as a threshold for CLNM.

The tumor stage is also a factor that may affect CLNM; in Gui's study in 2018, tumor gross extrathyroidal extension was found to be an independent predictor of CLNM [30]. These results correspond with our study that the T1b-T3 tumor group increased the likelihood of micrometastatic lymph nodes, with *p* < 0.05.

Several recent studies reported that young age can be a risk factor for CLN metastasis. Based on SEER database, a study aimed to investigate whether younger PTC patients were associated with higher metastatic lymph node rates conducted by Wang et al. in 2018; the results demonstrated that younger PTC patients have an increased predisposition for CLNM regardless of T stage, especially under 30 years old group [31]. In our study, with the cutoff is 29 years old, univariate logistic regression shows that under 29 years old patients group is associated with higher risk for CLNM with *p* = 0.03.

According to ATA 2015 and National Comprehensive Cancer Network (NCCN) 2022 guidelines, patients with five or fewer micrometastatic lymph nodes in postoperative pathology results can be classified as low-risk group and do not require further total thyroidectomy. However, for patients with a higher number of metastatic lymph nodes (more than five positive nodes), the necessity of completing a total thyroidectomy is still controversial [1, 32]. NCCN 2022 guidelines recommend performing a second total thyroidectomy in this group. On the other hand, in 2020, Choi et al. conducted a study on 876 patients with papillary thyroid microcarcinoma undergoing thyroid lobectomy surgery and prophylactic CND. These patients were divided into two groups based on CLN metastasis status: CLN-positive but do not have second total thyroidectomy surgery and CLN-negative groups. This study demonstrates that there is no statistically significant difference in the risk of recurrence or disease-free survival rates between the two groups. The median follow-up is 12.8 ± 4.3 (8.3–30.5) years and 13.4 ± 4.4 (8.3–32.3) years, respectively. According to these results, the authors concluded that the second total thyroidectomy surgery is not necessary in patients with positive CLN metastasis after the pCND [33]. Therefore, when the number of microscopic metastatic lymph nodes >5, patients can choose a second total thyroidectomy or an active surveillance strategy. However, the second surgery is accompanied by a higher risk of damage to RLN and parathyroid gland, and with patients who cannot or do not want to follow an active surveillance, surgeons should be aware of the risk factors for the possibility of high-volume lymph node metastases in order to make an appropriate decision for the extension of thyroidectomy to avoid the second operation.

With the cutoff value of the number of metastatic lymph node as 5, we separate patients with CLN metastasis into two groups: low metastatic lymph node volume group (≤5) and high metastatic lymph node volume group (>5); univariate analyses showed that tumor diameter on US and microscopic capsular invasion are not associated with high-volume CLNM with *p* > 0.05. Therefore, tumor size on ultrasound did not change the decision of surgeons to have a total thyroidectomy or only partial thyroidectomy in operation. The cause of this difference compared to the ATA 2015 recommendation can be explained that in our study, all patients had tumor diameter under 2 cm, while in ATA 2015 recommendation, total thyroidectomy was indicated in tumors with diameter larger than 4 cm [1, 32].

On the other hand, by using univariate and multipolarity analysis, we found that stage of tumor was significantly associated with high volume of CLNM with *p* < 0.05. But in the subgroup analysis, we found no significant difference in two groups T1a and T1b, with OR (95% CI) of 0.5 (0.1–5.1). Therefore, T1a and T1b patients may have a similar extend of thyroid resection. However, there is a statistically significant difference between two groups: noninvasive (T1a-T1b) and invasive tumor (T3b), when compared using the same criteria, with an OR (95% CI) of 0.1 (0.01–0.2).

Another factor that affects the number CLNM is the patient's age at operation. Young age not only is associated with a higher rate of CLN metastasis but also increases the number of metastatic lymph nodes; this was proven in several studies. In a large study on 2329 clinically N0 PTC patients in Asan Medical Center, Korea, between 2005 and 2012, Oh et al. found that high‐volume CLNM was more frequently found in younger patients [34]. Our result is similar: univariate and multivariate analyses demonstrated that with the cutoff age of 29 years old, younger patient is an independent factor of high number of CLNM with *p* < 0.05. Thus, we assume that patients with the T3b-gross tumor invasion and patients under 29 years of age have a higher rate and high volume of CLNM. Therefore, if these patients cannot follow an active surveillance strategy or are not willing to have the conservative procedure, indication to have total thyroidectomy is obvious.

## 5. Conclusion

In summary, CLNM rate in patients with cN0 PTC undergoing TOETVA procedure accounted for 39.9%. With the facilities of pCND by TOETVA, a procedure that is widely approved for early PTC and has excellent cosmetics and oncological results, pCND should be considered in patients with risk factors like young age or large tumor. High volume of CLNM is associated with young age and gross tumor extension, and total thyroidectomy should be indicated in these patient groups.

## Figures and Tables

**Table 1:** Clinicopathological characteristics of patients.

Variables	Value
Age (mean-min max)	36.1 ± 9.1 (13–67)
Gender	
Male	14 (4)
Female	332 (96)
Graves' disease	4 (1.15)
Tumor size on ultrasound	
Size of the tumor that is suspected cancer (mm)	7.6 ± 3.7 (2.6–20)
Cancer size	
Right/left lobe	329 (95.1)
Isthmus/both lobes	17 (4.9)
Procedure	
Total thyroidectomy	55 (15.9)
Conservative thyroidectomy	291 (84.1)
Central neck dissection	
One side	298 (86.1)
Both sides	48 (13.9)
Occult lymph node metastasis ratio	138 (39.9)
Number of lymph node (median–IQR)	
Harvested	
One side CND	5 (3–7)
Both side CND	7 (3–10)
Metastasis	
One side CND	2 (1–3)
Both side CND	3 (2–6)
The ratio of metastasis lymph node/harvested lymph node (*n* = 138)	0.5 ± 0.3 (0.05–1)
Tumor classification	
T1a	267 (77.2)
T1b	49 (14.2)
T3b	30 (8.6)
Postoperative pathology	
Pathology type	
Papillary carcinoma	340 (98.3)
Variants of papillary carcinoma	6 (1.7)
Microscopic capsular invasion	
No	256 (74)
Yes	90 (26)
Thyroiditis	
No	285 (82.4)
Yes	61 (17.6)

Number in parentheses represents percentage.

**Table 2:** Analysis of factors affecting the number of harvested lymph nodes.

Variables	No lymph node on surgery	With lymph node on surgery		Univariate	
	*n* = 10	*n* = 336		OR (95%)	*p*
Age					
<29	2 (20.0)	69 (20.5)		0.97 (0.2–4.7)	1
≥29	8 (80.0)	267 (79.5)			
Gender					
Male	0 (0)	14 (4.2)		—	1
Female	10 (100)	322 (95.8)			
Graves' disease history					
Yes	0	4 (1.2)		—	1
No	10 (100)	332 (918.8)			
Tumor size on US	9 ± 5.3 (4.3–20)		7.6 ± 3.6 (2.6–20)	0.5 (0.13–1.7)	0.4
Tumor location					
Single lobe	9 (90)	320 (95.2)		0.45 (0.05–3.8)	0.4
Both lobes/isthmus	1 (10)	16 (4.8)			
Surgical procedure					
Total thyroidectomy	2 (20)	53 (15.8)		0.7 (0.3–6.5)	0.7
Conservative thyroidectomy	8 (80)	283 (84.2)			
Central neck dissection					
Both sides	8 (80)	290 (86.3)		0.6 (0.13–3.1)	0.6
One side	2 (20)	46 (13.7)			
Tumor classification					
T1a	7 (70)	260 (77.2)		—	0.4
T1b	2 (20)	45 (13.6)			
T3b	1 (10)	31 (9.2)			
Microscopic capsular invasion					
Yes	3 (30)	87 (259)		0.8 (0.21–3.2)	0.7
No	7 (70)	249 (74.1)			
Thyroiditis	5.5 ± 0.2 (0–21)	8.3 ± 0.7 (1–24)		—	0.002

**Table 3:** Analysis of factors affecting metastatic status of lymph nodes.

Variables	Nonmetastatic nodes	Metastatic nodes	Univariate analysis	
	*n* = 208	*n* = 138	OR (95%)	*p*
Age				
<29	34 (16.3)	37 (26.8)	0.5 (0.3–0.9)	0.02
≥29	174 (83.7)	101 (73.2)		
Gender				
Male	7 (3.4)	7 (5.1)	0.6 (0.2–1.9)	0.6
Female	201 (96.6)	131 (94.9)		
Graves' disease				
No	2 (1)	2 (1.4)	0.7 (0.1–4.7)	0.7
Yes	206 (99)	136 (98.6)		
Tumor diameter on US (mm)	7.1 ± 3.3 (2.6–20)	8.5 ± 0.3 (2.7–20)	1.1 (1.0–1.2)	0.00
Tumor location				
Single lobe	200 (96.2)	129 (93.5)	1.7 (0.7–4.6)	0.3
Both lobes/isthmus	8 (3.8)	9 (6.5)		
Surgical procedure				
Total thyroidectomy	28 (13.5)	27 (19.6)	0.6 (0.4–1.1)	0.1
Conservative thyroidectomy	180 (86.5)	111 (80.4)		
Central neck dissection				
Both sides	24 (11.5)	24 (17.4)	0.6 (0.3–1.1)	0.12
One side	184 (88.5)	114 (28.6)		
Tumor classification				
T1a	169 (81.2)	98 (71)	1.8 (1.1–2.9)	0.03
≥T1b	39 (18.8)	40 (29)		
Microscopic capsular invasion				
No	153 (73.6)	103 (74.6)	0.9 (0.6–1.5)	0.82
Yes	55 (26.4)	35 (25.4)		
Thyroiditis				
No	168 (80.8)	117 (84.8)	0.8 (0.4–1.3)	0.3
Yes	40 (19.2)	21 (15.2)		

## Data Availability

The data used to support the findings of this study are included within the article.
